# Implementation of Virtual and Face-to-Face Childbirth Preparation Training for the Spouses of the Primiparous Women to Reduce the Fear of Childbirth, Improve the Pregnancy Experience, and Enhance Mother- and Father-Infant Attachment: Protocol for a Quasiexperimental Clinical Trial

**DOI:** 10.1155/2021/6686934

**Published:** 2021-04-11

**Authors:** Zari Doaltabadi, Leila Amiri-Farahani, Seyedeh Batool Hasanpoor-Azghady

**Affiliations:** ^1^Department of Reproductive Health and Midwifery, School of Nursing and Midwifery, Iran University of Medical Sciences, Tehran, Iran; ^2^Department of Reproductive Health and Midwifery, Nursing Care Research Center, School of Nursing and Midwifery, Iran University of Medical Sciences, Tehran, Iran

## Abstract

**Background:**

Men have a special role to play in promoting maternal and child health during pregnancy, childbirth, and postpartum period. The health of women also requires the participation and cooperation of men. The aim of this study is to compare the effect of virtual and face-to-face childbirth preparation training for spouses of the primiparous women on the pregnancy experience, fear of childbirth (FOC), and mother- and father-infant attachment.

**Methods:**

The primiparous women attending the prenatal clinics of Lolagar Hospital and Azadi and Tehransar health centers of Tehran along with their husbands will be studied. The inclusion criteria for the women's husbands are the first experience of becoming a father, being at least 18 years of age, and the ability to read and write. The exclusion criteria for women's husbands are the history of physical/mental illness; being a smoker; and consuming alcohol, drugs, or psychotropic substances. The participants will be selected by the convenience sampling method and will be divided into three groups of study A, study B, and control. Spouses in study groups A and B will receive childbirth training through virtual and face-to-face methods with similar content, respectively. The control group only receives ordinary prenatal care. At the 18–20 weeks of gestation, demographic information, pregnancy experience scale (PES), and version A of Wijma delivery expectancy/experience questionnaire (WDEQ-A) will be completed. At 37-38 weeks of gestation, the PES and WDEQ-A questionnaires will be completed again and maternal postnatal attachment scale (MPAS) and postnatal paternal-infant attachment questionnaire (PPAQ) will be completed by the parents 12 weeks after the delivery. *Discussion*. Improving the experience of pregnancy, especially reducing the FOC and creating a positive attitude towards it, is a vital strategy to promote vaginal childbirth and reduce the number of cesarean sections requested by women. Achieving this will reduce the cost of health care and improve the quality of life during pregnancy, after childbirth, and during the growth and development of infants. *Ethics and Dissemination*. This research has been funded by the Iran University of Medical Sciences, approved by the Thailand Registry of Clinical Trials, and will commence in May 2020. Results will be disseminated through peer-reviewed journals and shared with the academic and medical community to pregnancy and childbirth outcomes. This trial is registered with TCTR20200515011.

## 1. Introduction

Improving maternal health is one of the important goals of the Millennium Development Goals. Given that pregnancy is one of the most sensitive periods in any woman's life, and most deaths that occur during pregnancy or childbirth are preventable, providing health care during and after the pregnancy is one of the main strategies for promoting the health of mothers and infants and preventing their mortality [1]. Men's participation during pregnancy increases women's acceptance of prenatal care [2], which reduces the negative behaviors of women and improves the outcomes of pregnancy [3]. On the other hand, the lack of participation and support of spouses in prenatal care is associated with increased postpartum depression, lack of desire for breastfeeding, and anxiety and stress in women [4, 5]. Women who are dissatisfied with their husbands' support during pregnancy are at higher risk for having a negative experience of childbirth [6]. The support of the husband during pregnancy leads to an increased ability of women to tolerate pressures and difficulties of pregnancy and childbirth [7]. On the other hand, factors such as experiencing and counting fetal movements [8], listening to music, having massage [9], receiving support from the family, friends, and spouse, having mother-child skin contact, providing kangaroo care immediately after birth [10], and receiving pregnancy training have a positive effect on attachment [11].

Men need training and information to support their wives during pregnancy [12]. Today, it is recommended to provide educational programs for the family, especially the husband to increase maternal support during pregnancy [5]. This training increases men's knowledge, attitudes, and performance in maternal health care and also improves couples' relationships; meanwhile, in most countries, men's participation in prenatal care is not common [13]. In Iran, it is not common for the husbands to intervene in pregnancy-related issues, even though there is no legal or Sharia prohibition in this regard [14]. This training can help men to accept their role and adapt to it even though it is sometimes difficult for them. Assessing men's educational needs in prenatal care is necessary before conducting the training [15].

Many member states of the World Health Organization (WHO) have prioritized men's participation in prenatal care as well as women's empowerment in their reproductive health programs to reduce mortality and inequality and improve women's health, as these two have synergistic and positive effects on each other [16]. Despite significant evidence on the beneficial effects of male participation in prenatal care, it has not yet been effectively promoted globally [14].

The results of studies on men's participation in supporting their spouses show that the level of participation varies in different parts of the world. The results of a study by Aliabedian et al. in Babol showed that 69% of Iranian men participate in prenatal care [17]. Mortazavi et al. reported 25% of men participated in prenatal care in Sabzevar, Iran [14], which could indicate a difference in social and cultural factors in these two regions of Iran [5]. However, compared to developed countries, the participation of Iranian men in prenatal care is low [14]. In a qualitative study by Mortazavi and Mirzaii to assess the causes, barriers, and outcomes of men's participation in pregnancy and childbirth care programs, their lack of knowledge about pregnancy, their workload, and the feminine atmosphere of health care centers were among the obstacles to men's participation in prenatal care [18].

By using new educational systems such as e-learning, we can overcome educational problems such as lack of time and special place for education and the need for experienced and trained people for education. E-learning can solve some of the problems that exist in our country in the field of childbirth preparation courses through its interactive, self-guiding, and flexible nature [19]. It is also essential to use different educational methods for men's participation in prenatal care [18]. Men's training programs are more successful if they are supported by the mass media, NGOs, and public education and are tailored to men's needs [15]. Considering the obstacles mentioned for men's participation in prenatal care and also the ease of access to the Internet and social media in Iran, the present study aims to compare the effect of virtual and face-to-face childbirth preparation training for spouses of the primiparous women on the pregnancy experience, fear of childbirth (FOC), and mother- and father-infant attachment.

### 1.1. Outcomes

The primary outcome of the present study is the pregnancy experience. The secondary outcome of the present study is FOC. In addition, the third and fourth outcomes are the mother- and father-infant attachment.

### 1.2. Hypothesis

The primary hypothesis of the present study is pregnancy experience score in the two intervention groups will be more positive from the control group. The secondary hypothesis in this study is the lower level in the fear of childbirth score in the two intervention groups compared to the control group. The third and fourth hypotheses are higher level in the score of parental attachment in the two intervention groups compared to the control group.

## 2. Materials and Methods

### 2.1. Setting

Data were collected in the prenatal clinics of Lolagar Hospital (study group A), Azadi health center (study group B), and Tehransar health center (control group) in Tehran, Iran, where the childbirth preparation courses are being offered in accordance with the educational programs of the Ministry of Health with high quality and in regular basis. It should be noted that the courses are held in groups of 10–12 people in the above-mentioned centers.

### 2.2. Inclusion and Exclusion Criteria

All 18–35-year-old pregnant women, who will be referred to the prenatal clinics of Lolagar Hospital as well as Tehransar and Azadi health centers and are eligible to receive prenatal care during the 18 and 20 weeks of gestation, will be asked to participate in the study. Inclusion criteria for women are to have Iranian nationality, be in gestational age of 18–20 weeks, have the ability to read and write, have single and low-risk pregnancy, and participate in childbirth preparation classes. Also, exclusion criteria for women are the history of infertility and physical/mental disease, being a smoker, and consuming alcohol, drugs, or psychotropic substances. The inclusion criteria for the women's husbands are the first experience of becoming a father, at least 18 years of age, Iranian nationality, the ability to read and write, and possession of smartphones or computers with access to the Internet and Telegram application until the end of the study. The exclusion criteria for women's husbands are the history of physical/mental illness and being a smoker, consuming alcohol, drugs, or psychotropic substances.

After the sampling, exclusion criteria would include preterm delivery or any symptoms of high-risk pregnancy, hospitalization in the neonatal intensive care unit, being absent for more than two sessions of childbirth preparation classes, and reluctance to continue with the study. In addition, the men of the study group A who do not send any feedback through Telegram application and the men of the study group B who do not attend more than one preparation sessions will be excluded from the study.

### 2.3. Research Design and Sampling

**Figure 1:**
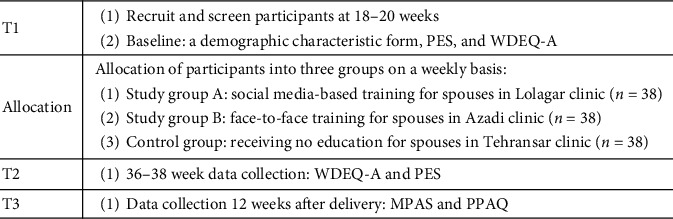
Key points for data collection.

This is a quasiexperimental study with a control group (parallel design) that will be performed on 114 primiparous women. Sampling will be done in three health centers. To complete the sampling in group A, the researcher will refer to Lolagar Health Center. Due to the presence of more husbands of nulliparous women in childbirth preparation classes, Azadi health center was allocated to complete the sampling process in group B, and finally Tehransar health center will be to complete the control group. Participants will be equally and not randomly divided into two study groups of A and B and one control group (Figure 1). Spouses of the participants in study groups A and B will be trained virtually and face-to-face, respectively. Also, the spouses in the control group will receive no education regarding childbirth preparation during pregnancy.

Based on the study's objectives, a Telegram channel called: “Virtual childbirth preparation courses for fathers” will be created for uploading the educational content. The educational contents will be provided based on the standard regulations of Iran.

To increase the quality of training and prevent sending the whole content at once, the content of each session will be provided in sections that will be uploaded at a specific time. The educational contents will include five videocasts, four PDF files, and eight video files, which are shown in Table 1.

To ensure that the messages sent on the Telegram social media are read by the participants, before joining the group, they will be asked to share their last seen status with the researcher. Moreover, they will be required to become online at least once a day to watch the videos or read the messages. The last seen time of the participants will be controlled by the researcher, and those who are not online, do not see messages, or do not give feedback for seven days are initially contacted through message. In case of receiving no answer, the researcher will contact them through a phone call to ask for the reason. If needed, another cell phone number will be obtained to resend a message by Telegram social media to that number. Otherwise, the participant will be excluded from the study.

On the other hand, participants in the study group B will receive face-to-face training, and the content of the first and third sessions will be provided to the spouses in the form of an educational booklet. The contents of the second and fourth sessions will be based on the third and eighth sessions of childbirth preparation classes at the Azadi health center with the presence of spouses. The teaching will be performed in the form of a lecture by the midwife using teaching aids for 90 minutes. Educational content will be taught in accordance with men's educational needs to participate in childbirth preparation classes reviewed by Simbar et al. [15]. Table 1 presents the time, content, and objectives of the educational courses that are similar for both study groups. To increase the generalizability of the results, another group will be considered as the control group, the members of which will receive no educational courses about pregnancy and childbirth preparation whether through social media or face-to-face. It is important to note that all three groups will receive ordinary perinatal care. In addition, all participants will be followed up until 12 weeks after delivery.

### 2.4. Outcomes and Data Collection

The primary outcome of the present study is the pregnancy experience measured by the brief version of the pregnancy experience scale (PES) [20]. Version A of the Wijma delivery expectancy/experience questionnaire (WDEQ-A) measures the level of FOC as the secondary outcome [21]. In addition, the third and fourth outcomes are the mother- and father-infant attachment which will be measured by the MPAS [22] and PPAQ [23], respectively. Data collection is shown in Figure 1. Data will be collected at 3 time points via self-report and Telegram social media messages: recruitment and screening of the participants (demographic characteristics questionnaire, PES, WDEQ-A) ≈ 20–24 weeks (T1); 37-38 weeks (PES and WDEQ-A), (T2); and 12 weeks after delivery (MPAS and PPAQ) (T3).

### 2.5. Sample Size

In the present study, recruitment will be done by convenience method at respective health centers in 18–20 weeks of pregnancy. The sample size was determined based on *α* = 0.05, *β* = 0.2, and a dropout rate of 10% for all of outcomes will be assessed during the current study. For primary outcome, the pregnancy experience, an estimated standard deviation of 7.5, and an accuracy of 7 (equal to the estimated standard deviation) were considered. The sample size was estimated to be 18 participants per group. For secondary outcome, fear of childbirth, the sample size was calculated to consider *δ*_1_ = 25.1, *δ*_2_ = 18.9 based on the study by Serçekuş and Başkale, 2015 [24], *d* = 15 (difference between the mean score of fear of childbirth after the intervention between face-to-face and virtual groups), and 35 participants per group have been estimated. Considering a dropout rate of 10% during the study, we aim to recruit 38 participants per group. For the third and fourth outcomes, mother- and father-infant attachment, the sample size was determined based on effect size (ES) = 0.7, and 10% possibility of dropouts (*n* = 35 participants in each group). Because the fear of childbirth variable was higher, this variable was used to determine the sample size.

### 2.6. Data Collection

The demographic characteristics questionnaire consists of four sections. The first part deals with individual characteristics of the pregnant women such as age, level of education, employment status, economic level, and mode of delivery at birth. The second part is related to the spouse's personal profile including age, level of education, and employment status. The third part covers the history of pregnancy (the date of the last menstruation period, the expected date of delivery, and the recent pregnancy status), and the fourth part includes four questions related to the spouse's support of women during pregnancy.

The brief version of PES was first developed by DiPietro (2008) with 20 items. The first and second 10 items assess the uplifts and hassles among pregnant women. The items are scored based on a 4-point Likert scale ranging from not at all (0) to somewhat (1), quite a bit (2), and a great deal (3). The sum scores for each section are within the range of 0–30, and the higher scores indicate uplifts and hassles. The reliability of the tool was measured using internal consistency and is estimated at 0.82 and 0.83 Cronbach's alpha for uplifts and hassles, respectively [20]. The reliability was also measured using the test-retest method for consistency across time and the results were 0.56–0.83. In Iran, the Persian translation of this tool was carried out by Hajifoghaha while its psychometric properties were determined by Ebadi. The face, content, and construct validity of PES have also been confirmed. In order to determine the reliability of the test, an internal consistency with a Cronbach alpha of 0.777 and 0.672 was used for uplifts and hassles, respectively. Moreover, in this regard, intraclass correlation coefficients of 0.712 and 0.672 were used for uplifts and hassles, respectively. This tool is valid in weeks 15–38 of gestation [25].

The WDEQ-A was developed by Wijma (1998) and consists of 33 items that are scored based on a 6-point Likert scale ranging from “not at all…” to “extremely….” The total scores were obtained within the range of 0–165 so that the scores of ≤37, 38–65, 66–84, and ≥85 were recognized as mild, moderate, severe, and clinical FOC, respectively. Notably, reverse scoring was applied to items 2, 3, 6, 7, 8, 11, 12, 15, 19, 20, 24, 25, 27, and 31 [21]. The reliability of the results during the last trimester has been estimated at 0.89 Cronbach's alpha, and the split-half reliability of the test has been obtained at 0.91 [21]. The Persian version of this questionnaire was developed by Abedi et al. in Iran, and the reliability of this scale has been reported at 0.64 Cronbach's alpha for the whole pregnancy period [26].

The MPAS was designed in 1998 by Condon and Corkindale, which includes 19 items, with a high score indicating a mother's higher attachment to the infant. In this questionnaire, the sum of all scores in the three subscales reveals the total score of attachment. The three subscales include the quality of attachment, absence of hostility, and pleasure in interaction. The subscale of attachment quality includes 9 items (3, 4, 5, 6, 7, 10, 14, 18, and 19), in which the lowest score is 5 and the highest score is 45. The subscale of absence of hostility includes 5 items (1, 2, 15, 16, and 17) which have the lowest score of 5 and the highest score of 25. The subscale of pleasure in interaction also includes 5 items (8, 9, 11, 12, and 13), which has the lowest score of 5 and the highest score of 25. The options for the different items in this scale vary, and, in some cases, the items have five options, some others have four options, and some have two options. In five-option items, the range of scores for each item is from five to one, so that the person with the highest attachment gets higher scores. In the four-option items, the scores are (1, 2.3, 3.6, and 5). In the two-option items, the scores are in the form of 1.5. The internal consistency in a study of 200 mothers of 6-month-old children has been determined with the Cronbach's alpha of 0.78. The reliability was also measured using the test-retest method for consistency across time and the results were 0.86 [22]. This questionnaire has been translated by Dezvaree et al. Internal consistency in study has been determined with the Cronbach's alpha of 0.9 [27].

PPAQ was designed by Condon et al. It has 19 items in three subscales of patience and tolerance that include 8 items (1, 2, 6, 11, 13, 17, 18, and 19), the pleasure in interaction that includes 7 items (4, 5, 8, 9, 10, 12, and 15), and affection and pride that include 4 items (3, 7, 14, and 16). These items are scored according to specific instructions. Scoring in the items of 5, 10, 12, and 14 is done with a 5-point Likert's scale from 5 to 1 so that item one receives a score of 5, and item five receives a score of 1. Items 17, 18, and 19 are based on the 4-option Likert's scale so that item one receives a score of 1, item two receives a score of 2.3, item three receives a score of 3.6, and item four receives a score of 5. In the items 9, 11, and 15, scoring is based on the four-option Likert's scale with item one receiving a score of 5, item two receiving a score of 3.6, item three receiving a score of 2.3, and item four receiving a score of 1. In items 8 and 13, item one gets a score of 5 and item two gets a score of 1. Also in item 16, item one receives a score of 5, item two gets a score of 3, and item three receives a score of 1. The range of scores in the questionnaire is between 19 and 95, and higher scores indicate higher attachment. In the sixth month, Cronbach's alpha was found to be 0.81, and, in the 12th month, it was 0.78 [23]. This questionnaire has been translated in Iran by Arshadi Bostanabadi et al., and its internal consistency with Cronbach's alpha was confirmed to be 0.86 [10].

### 2.7. Data Analysis

The collected data will be analyzed in SPSS software (version 19) using descriptive statistics, such as frequency distribution tables. Moreover, the Chi-square test and analysis of covariance (ANCOVA) will be utilized to compare the quantitative and qualitative variables in the three groups, respectively. In addition, the paired *t*-test will be employed within group comparison. The *p* value of less than 0.05 will be considered statistically significant.

### 2.8. Patient and Public Involvement

Participants were not involved in the development of this research. However, the overall results of the study will be communicated to the study participants by sending the final publication (article) to the provided e-mail address.

### 2.9. Ethical Considerations

The protocol of the present study has been approved by the Ethics Committee of Iran University of Medical Sciences, Tehran, Iran (IR.IUMS.REC.1398.325) and also has been registered in the Thailand Clinical Trial Registration Center (TCTR) (TCTR20200515011). All participants will be fully informed about the objectives and process of the study and will provide a written informed consent. The information obtained during the study process will remain confidential. Women and their spouses will be told that they can withdraw from the study at any time. During the study process, no costs will be imposed on the samples and all services will be completely free. In the present study, only one code will be written in the questionnaires and all information related to the pregnant women will be entered into the information software anonymously. It should be noted that the researcher and all instructors of childbirth preparation classes will participate in a 60-hour teaching course organized by the Ministry of Health and will obtain a certificate for teaching childbirth preparation.

## 3. Results

This study was funded by Iran University of Medical Sciences in June 2019 and will be approved at the Thailand Clinical Trial Registration Center in May 2020. Data collection in this study is projected to take place from May 2020 to January 2021. Data analysis will be conducted in February 2021 and it is expected that the paper is to be published by April 2021.

## 4. Discussion

This quasiexperimental clinical trial with the control group aims to compare the effect of virtual and face-to-face childbirth preparation training for spouses of the primiparous women on the pregnancy experience, FOC, and mother- and father-infant attachment. This study will be conducted by the Reproductive Health Department of the School of Nursing and Midwifery of Iran University of Medical Sciences, at the prenatal clinics of Lolagar Hospital and Azadi and Tehransar health centers. The main results of this study will help to improve the experience of pregnancy, reduce the FOC in pregnant women, and enhance the mother- and father-infant attachment.

To our knowledge, there are no published studies on the effect of two different educational methods on primiparous husbands on pregnancy experience, fear of childbirth, and parental attachment to the infant. This proposed project examines an educational intervention whose content is based on a qualitative study that investigates the educational needs of men to participate in prenatal care [15]. E-learning can solve some of the problems that exist in our country in the field of childbirth preparation courses through its interactive, self-guiding, and flexible nature. It is hoped that in such educational intervention two different educational methods will produce more favorable health outcomes. The use of new educational methods such as mobile software shows more desire of participants to participate in training classes. As a result, the strengths of this study will be the key to potential success. If e-learning can have the same effect on the outcomes of this study as face-to-face training, this method can be a useful way to increase the participation in childbirth preparation classes for those men who cannot attend childbirth preparation classes in person. If successful, the intervention may be adopted in other populations with other outcome and randomized clinical trial study designs. By using new educational systems such as e-learning, we can overcome educational problems such as lack of time and special place for education, and the need for experienced and trained people for education. Also, a range of outcomes relating to men's involvement in childbirth preparation courses will be assessed.

The proposed study has a number of potential limitations. First, there is only a limited time in which the participants can be recruited. Second, due to the low attendance of men in childbirth preparation classes in many health centers, it is not possible to perform random allocation of participants in the groups. As a result, due to the nature of educational interventions, blinding of the participants is not possible in this study.

## Figures and Tables

**Table 1:** The educational content of social media-based training group and face-to-face childbirth preparation courses group.

Time	Content	Objectives	Uploading the virtual content to the Telegram channel
First session: 24–28 gestational week	(1) Pregnancy diet	(1) Pregnancy diet with emphasis on what to eat (2) An introduction to the food pyramid	1 videocast and 2 video files for theoretical content
Second session: 28–30 gestational week	(1) Mental health during pregnancy	(1) An introduction to the fetal growth and development (2) Preparing for motherhood (3) Preparing for fatherhood	2 videocasts, 1 PDF, and 2 video files for theoretical content
Third session: 32-33 gestational week	(1) Planning for delivery and selecting the type of delivery	(1) Natural delivery vs. cesarean section (2) Different pain control methods during labor (3) Selecting the delivery location and necessary equipment for delivery	2 videocasts , 1 PDF, and 2 video files for theoretical content
Fourth session: 37 gestational week	(1) Neonatal care	(1) Neonatal care and risk factors	2 PDF files and 2 videos file for theoretical content

## Data Availability

Access to data is restricted.

## Abbreviations

PES: Pregnancy experience scale
WDEQ-A: Wijma delivery expectancy/experience questionnaire-A
MPAS: Maternal postnatal attachment scale
PPAQ: Postnatal paternal-infant attachment questionnaire
TCTR: Thailand clinical trial registration
FOC: Fear of childbirth
ANOVA: Analysis of variance
WHO: World Health Organization.

## Supplementary Materials

Supplementary Materials This study was performed according to the Standard Protocol Items: Recommendations for Interventional Trials (SPIRIT) statement in supplemental file.

## References

[1] Yan J. (2017). The effects of prenatal care utilization on maternal health and health behaviors. *Health Economics*.

[2] Uldbjerg C. S., Schramm S., Kaducu F. O., Ovuga E., Sodemann M. (2020). Perceived barriers to utilization of antenatal care services in northern Uganda: a qualitative study. *Sexual & Reproductive Healthcare*.

[3] Kaye D. K., Kakaire O., Nakimuli A., Osinde M. O., Mbalinda S. N., Kakande N. (2014). Male involvement during pregnancy and childbirth: men’s perceptions, practices and experiences during the care for women who developed childbirth complications in Mulago Hospital, Uganda. *BMC Pregnancy and Childbirth*.

[4] Azimi M., Fahami F., Mohamadirizi S. (2018). The relationship between perceived social support in the first pregnancy and fear of childbirth. *Iranian Journal of Nursing and Midwifery Research*.

[5] Zamani P., Ziaie T., Lakeh N. M., Leili E. K. (2019). The correlation between perceived social support and childbirth experience in pregnant women. *Midwifery*.

[6] Bryanton J., Gagnon A. J., Johnston C., Hatem M. (2008). Predictors of women’s perceptions of the childbirth experience. *Journal of Obstetric, Gynecologic & Neonatal Nursing*.

[7] Sun Y.-C., Hung Y.-C., Chang Y., Kuo S.-C. (2010). Effects of a prenatal yoga programme on the discomforts of pregnancy and maternal childbirth self-efficacy in Taiwan. *Midwifery*.

[8] Sajjadi Anari S., Zahrakar K., Mohsenzadeh M., Karamnia M., Shokoohi Yekta M., Alavinezhad S. (2016). Efficacy of maternal fetal attachment techniques on enhancing mother’s attachment to the fetus. *Journal of Developmental Psychology*.

[9] Azogh M., Shakiba M., Navidian A. (2018). The effect of cognitive behavioral training on maternal-fetal attachment in subsequent pregnancy following stillbirth. *Hayat*.

[10] Arshadi Bostanabadi M., Valizadeh S., Rezanezhad J., Jabbari T. (2014). Paternal–newborn bonding and its related factors. *Nursing and Midwifery Journal*.

[11] Parsa P., Saiedzadeh N., Roshanai G., Masoumi S. Z. (2016). The effect of training on maternal-fetal attachment (MFA) in nulliparous women: a randomized clinical trial. *Scientific Journal of Hamedan Nursing and Midwifery Faculty*.

[12] Kululanga L. I., Sundby J., Chirwa E. (2011). Striving to promote male involvement in maternal health care in rural and urban settings in Malawi-a qualitative study. *Reproductive Health*.

[13] Jennings L., Na M., Cherewick M., Hindin M., Mullany B., Ahmed S. (2014). Women’s empowerment and male involvement in antenatal care: analyses of Demographic and health surveys (DHS) in selected African countries. *BMC Pregnancy and Childbirth*.

[14] Mortazavi F., Delara M., Akaberi A. (2014). Male involvement in prenatal care: impacts on pregnancy and birth outcomes. *Nursing And Midwifery Journal*.

[15] Simbar M., Nahidi F., Tehran F. R., Ramezankhani A. (2010). Fathers’ educational needs for perinatal care in urban Iran: a qualitative approach. *Journal of Biosocial Science*.

[16] Fathnezhad Kazemi A., Sharifi N., Simbar M. (2017). A review on different aspects of men’s participation in antenatal care. *Jorjani Biomedicine Journal*.

[17] Aliabedian A., Agajani Delavar M., Khan Mohammmadi A. (2015). Iranian men’s attendance in pregnancy. *Caspian Journal of Reproductive Medicine*.

[18] Mortazavi F., Mirzaii K. (2012). Reason of, barriers to, and outcomes of husbands’ involvement in prenatal and intrapartum care program based on midwives’ experiences: a qualitative study. *Journal of Arak University of Medical Sciences*.

[19] Hamzekhani M., Hamidzade A., Vasegh Rahimparvar S., Montazeri A. (2014). Effect of computerized educational program on self-efficacy of pregnant women to cope with childbirth. *Journal of Knowledge & Health*.

[20] DiPietro J. A., Ghera M. M., Costigan K., Hawkins M. (2004). Measuring the ups and downs of pregnancy stress. *Journal of Psychosomatic Obstetrics & Gynecology*.

[21] Wijma K., Wijma B., Zar M. (1998). Psychometric aspects of the W-DEQ; a new questionnaire for the measurement of fear of childbirth. *Journal of Psychosomatic Obstetrics & Gynecology*.

[22] Condon J. T., Corkindale C. J. (1998). The assessment of parent-to-infant attachment: development of a self-report questionnaire instrument. *Journal of Reproductive and Infant Psychology*.

[23] Condon J. T., Corkindale C. J., Boyce P. (2008). Assessment of postnatal paternal-infant attachment: development of a questionnaire instrument. *Journal of Reproductive and Infant Psychology*.

[24] Serçekuş P., Başkale H. (2016). Effects of antenatal education on fear of childbirth, maternal self-efficacy and parental attachment. *Midwifery*.

[25] Hajifoghaha M., Ebadi A., Kariman N. (2016). Persian translation of the pregnancy experience scale (PES)–brief version: confirmatory factor analysis. *Methods*.

[26] Abedi P., Hazeghi N., Afshari P., Fakhri A. (2016). The validity and reliability of Persian version of Wijma delivery expectancy/experience questionnaire (version a) among Iranian nulliparous women. *Global Journal of Health Science*.

[27] Dezvaree N., Alaeekarahroudi F., KhanaliAgan L., TalebiGhane E. (2016). The mother-newborn s attachment and its related factors in mothers of hospitalized preterm neonates. *Health Care*.

